# Comparison of the Bacterial and Fungal Communities and Metabolic Functions of Cottonseed Hull Waste Compost Associated with High and Low Yields of Straw Mushroom *Volvariella volvacea*

**DOI:** 10.3390/microorganisms13020437

**Published:** 2025-02-17

**Authors:** Pattana Kakumyan, Lin Yang, Shunjie Liu, Changxia Yu, Zhengpeng Li, Mingjie Chen, Siam Popluechai, Yan Zhao

**Affiliations:** 1 Institute of Edible Fungi, Shanghai Academy of Agricultural Sciences, Shanghai 201403, China; pattana.kak@mfu.ac.th (P.K.); ylin_jade@163.com (L.Y.); jasonliu86@foxmail.com (S.L.); ycx41529@163.com (C.Y.); lizp_ln@126.com (Z.L.); mjfungi@126.com (M.C.); 2 School of Science, Mae Fah Luang University, Chiang Rai 57100, Thailand; siam@mfu.ac.th; 3 Microbial Products and Innovations Research Group, Mae Fah Luang University, Chiang Rai 57100, Thailand; 4 Gut Microbiome Research Group, Mae Fah Luang University, Chiang Rai 57100, Thailand

**Keywords:** straw mushroom, cotton waste compost, microbial diversity, physicochemical properties, biological efficiency

## Abstract

*Volvariella volvacea* was grown on cottonseed hull waste compost and divided into high-yield (HBE) and low-yield (LBE) conditions. Gene sequencing was used to examine bacterial and fungal populations during cottonseed husk waste composting. At the end of fermentation, the dominant bacterial genera in the HBE compost were *Chelatococcus* and *Thermobacillus,* while *Symbiobacterium* and *Acinetobacter* were more abundant in the LBE compost. *Ascomycota* and *Basidiomycota* dominated all the composting phases. The *Ascomycota* genera *Colletotrichum*, *Pichia*, *Mycothermus*, and *Thermomyces* dominated in phase II of HBE composting. The LBE compost had higher abundances of the *Basidiomycota* genera *Cystofilobasidium* and *Cryptococcus* than the HBE compost. The predicted pathotroph and saprotroph-symbiotroph abundances were more positively linked to HBE composting phase II than to LBE composting. High-biological-efficiency microbial communities are characterized by high pH, carbon, and nitrogen levels. Changes in physiochemical traits, microbial diversity, and metabolism affect the *V*. *volvacea* yield.

## 1. Introduction

Mushroom cultivation is crucial for providing nutrition to people globally. In 2020, the worldwide production of mushrooms and truffles was estimated to be 42 million tons [1]. China is the top producer of edible mushrooms [1,2]. The straw mushroom, with the scientific name *Volvariella volvacea* (Bull.: Fr.) Singer, is a significant mushroom variety cultivated for commercial purposes in China [3,4]. Abiotic variables such as temperature, pH, oxygen levels, moisture, and substrate composition play crucial roles in the growth and development of fruiting bodies. *V. volvacea* grows on a variety of substrates, with rice straw and cotton waste being the most commonly utilized materials for commercial cultivation. Compared to raw material, substrate fermentation cultivation is a widely used method due to the advantages of low pollution, low cost, simple process, and high economic benefits [5]. Factors such as the C/N ratio, temperature, moisture, and oxygen levels have a significant impact on the maturity of fermented substrates [6]. The microbial consortium is also involved in the fermentation process, which provides the mushroom with its primary source of nutrition [7].

Several publications have reported the role of microbial dynamics during substrate fermentation in the production of selective media for *Agaricus* [8,9,10,11,12] and *Pleurotus* species [5,13]. Differences in the composition of raw materials lead to variations in microbial communities, dominant species and their ecological functions [14,15]. Analysis of metabolic pathways and dynamic changes in the microbiota during fermentation is essential for establishing relationships among substrate properties, microbial communities, and their associated metabolic potential [5,16]. Rice straw [15,17], corncob [5], wheat straw [11,12], sugarcane straw [18], maize straw [19,20], and sugarcane bagasse [21,22] composting have been used for examining these relationships. Nonetheless, little information is available about the changes in microbial communities during cottonseed hull waste (CW) composting [23]. Even less is known about the relationships among physiochemical characteristics, microbial communities, and their metabolic potential for *V*. *volvacea* cultivation [24]. Although bacterial and fungal diversity during composting has been investigated for decades using high-throughput sequencing, the results are not sufficient to provide a comprehensive link between the structure and diversity of microbial communities and biological efficiency. An understanding of the microbial communities associated with certain production conditions and yields would significantly contribute to improving the efficiency of composts for enhancing mushroom productivity. Therefore, understanding microbial diversity, metabolic potential, and physiochemical properties is particularly important for finding the key factors affecting the cultivation and efficient production of *V*. *volvacea*.

Biological efficiency is an important parameter used to calculate the effectiveness of mushroom production [18,25]. The contribution of the microbes that affect the success of mushroom production by providing high biological efficiency is useful for improving productivity. Therefore, in this study, comparisons of associated bacterial and fungal community profiles and functional analyses of genes involved in composting under two different composting conditions (high (HBE) and low (LBE) biological efficiency conditions) were performed. 16S rRNA and 18S rRNA gene sequencing technology and functional predictions were used to analyze the microbial communities and their associated functions in the two composting conditions. In addition, the relationship of physicochemical properties with microbial communities and microbial metabolism will be discussed. These results will provide references which elucidate the potential functions on the dominant genera of cotton hull waste composting and will be of great value for metabolomics research of composting.

## 2. Materials and Methods

### 2.1. Mushroom Strain and Substrate Material

CW was purchased from local stores in Zhenjiang, Jiangsu Province, China. *V. volvacea* (strain V23) was obtained from the Institute of Edible Fungi, Shanghai Academy of Agricultural Sciences, Shanghai, China, and maintained on potato dextrose agar at 32 °C.

### 2.2. Compost Preparation and Sampling

All compost-associated experiments were performed indoors at the Institute of Edible Fungi, Shanghai Academy of Agricultural Science, Shanghai, from August to September 2019. The composting formula included 95% CW and 5% lime. The CW was soaked in lime water, and the initial moisture content was adjusted to approximately 70% (prewetting the substrate material, build heap (BH) or phase 0 (P0)). The substrate pile was fermented for 24 h, turned over, and continued to ferment for 24 h (1st fermentation, PI). The fermented substrate was stacked into a bed frame with a thickness of 10–15 cm, covering an area of 8 m^2^. The room temperature was set to 60–65 °C, and the samples were maintained for 10–12 h for fermentation (2nd fermentation, PII). After fermentation, the window was opened for ventilation, and the substrate was allowed to cool naturally to 35–40 °C. Mature compost was used for *V*. *volvacea* cultivation. The compost materials for sequencing were collected from P0, PI, and PII at a depth of 5 cm below the surface, employing a five-point sampling method, with 1 kg of sample taken from each point. The physicochemical properties and lignocellulosic enzymes were previously analyzed and reported by Liu et al. [26].

Mushroom fruiting bodies at the egg shape were harvested manually and weighed. Biological efficiency values (%BE) (fresh weight of fruiting bodies × 100/dry weight of substrate) for six replicates were calculated. Four mushroom houses in production were randomly followed up. The batches with the lowest and highest biological efficiency were chosen as representative samples and were designated as LBH and HBH, respectively.

### 2.3. DNA Extraction, PCR Amplification, and Sequencing

Total genomic DNA from the compost samples was extracted using the FastDNA^®^ SPIN Kit for soil (MP Bio, Irvine, CA, USA) according to the manufacturer’s instructions. DNA purity was assessed on a 1% agarose gel. The V4 hypervariable region of the 16S rRNA gene was amplified using the specific barcode primers 515F (5′–GTGCCAGCMGCCGCGGTAA–3′) and 806R (5′–GGACTACHVGGGTWTCTAAT–3′) to study bacterial diversity [24]. The fungal-specific barcode primer pair ITS5-1737F (5′–GGAAGTAAAAGTCGTAACAAGG–3′) and ITS2-2043R (5′–GCTGCGTTCTTCATCGATGC–3′) was used to amplify the internal transcribed spacer 1 (ITS1) region to study fungal diversity [27]. All PCRs were carried out with Phusion^®^ high-fidelity PCR Master Mix (New England Biolabs, Ipswich, MA, USA). The PCR products were purified with the GeneJET™ Gel Extraction Kit (Thermo Scientific, Waltham, MA, USA). Sequencing libraries were subjected to Illumina MiSeq sequencing at Novogene, Beijing, China.

### 2.4. Data Processing and Analysis

The single-end reads were assigned to samples based on their unique barcode and were truncated by removing the primer and barcode sequences. To obtain high-quality sequence data, quality filtering of the raw reads was performed using specific filtering conditions according to Cutadapt (V1.9.1, http://cutadapt.readthedocs.io/en/stable/, accessed on 10 March 2019) [28]. The output reads were then compared with a reference database (SILVA database, https://www.arb-silva.de/, accessed on 10 March 2019) [29] using the UCHIME algorithm (http://www.drive5.com/usearch/manual/uchime_algo.html, accessed on 10 March 2019) [30] to detect chimera sequences, and the chimera sequences were then removed to finally obtain the clean reads [31].

Sequence analysis of all filtered reads was performed using UPARSE software (UPARSE v7.0.1001, http://drive5.com/uparse/, accessed on 24 March 2019) [32]. Sequences with ≥97% similarity were assigned to the same operational taxonomic unit (OTU). A representative sequence from each OTU was screened for further annotation. Each OTU representative sequence was classified at six taxonomic levels (phylum, class, order, family, genus, and species) using the BLAST method in QIIME software (version 1.9.1, http://qiime.org/scripts/assign_taxonomy.html, accessed on 24 March 2019) [33] and the Unit (v7.2) database (https://unite.ut.ee/, accessed on 24 March 2019) [31]. The SILVA database (version 132) (https://www.arbsilva.de/) was used to annotate the taxonomic information [29]. To study the phylogenetic relationships of different OTUs and to compare the dominant species among the different samples (groups), MUSCLE software (V5, http://www.drive5.com/muscle/, accessed on 30 March 2019) was used for multiple sequence alignment [34]. The OTU abundance information was normalized using a standard sequence number corresponding to the sample with the fewest sequences. Subsequent analyses of alpha diversity and beta diversity were all performed based on the normalized output data.

Alpha diversity was used to analyze the complexity of species diversity per sample by employing 5 indices (observed species, Chao1, Shannon, Simpson, and Good’s coverage indices). All these indices were determined with QIIME (version 1.7.0) and visualized with R software (version 2.15.3). Two indices were selected to determine community richness: the Chao index (http://www.mothur.org/wiki/Chao, accessed on 17 April 2019) and the ACE index (http://www.mothur.org/wiki/Chao, accessed on 17 April 2019). Two indices were used to determine community diversity: the Shannon index (http://www.mothur.org/wiki/Shannon, accessed on 17 April 2019) and the Simpson index (http://www.mothur.org/wiki/Simpson, accessed on 17 April 2019). Additionally, one index was used to characterize the sequencing depth: Good’s coverage (http://www.mothur.org/wiki/Coverage, accessed on 17 April 2019).

Beta diversity analysis was employed to assess the variations in species composition among different samples. The beta diversity from both the weighted and unweighted UniFrac distances was calculated with QIIME software (version 1.9.1), and a UPGMA sample cluster tree was constructed. Principal component analysis (PCA) and nonmetric multidimensional scaling (NMDS) diagrams were generated to visualize the principal coordinates from complex multidimensional data using WGCNA, stats, and the ggplot2 package in R software for PCoA and the vegan package of R software for NMDS analysis. Comparisons of the relative abundances between groups at various classification levels were performed with Metastat to obtain *p* values, and then the *p* values were corrected to obtain *q* values using the Benjamini–Hochberg procedure [35]. The Spearman correlation coefficient of species and environmental factors was assessed using the curtest function in the psych package of R software, and its significance was tested. Then, the heatmap function in the heatmap package was used for visualization.

The functional annotations of the unigenes were made using BLASTN analysis with Kyoto Encyclopedia of Genes and Genomes (KEGG, http://www.kegg.jp/kegg/, accessed on 24 April 2019). An abundance cluster heatmap analysis of KEGG ortholog functions and a comparative analysis of metabolic pathways were performed based on an abundance table of each taxonomic hierarchy or functional abundance. Fungal ITS Extractor 2.0 was used to obtain the extracted sequences covering the ITS1 region. The fungal sequences were checked for chimeras using the method used for the bacterial sequences. The taxonomic classification was based on the fungal UNITE database [36], and OTUs containing fewer than 10 sequences were discarded. The fungal OTUs were transformed into text format, and the text was uploaded to FUNGuild: Taxonomic Function (http://www.funguild.org/, accessed on 24 April 2019) for fungal functional prediction [37].

## 3. Results and Discussion

### 3.1. Diversity and Compositions of Microbial Communities in LBE and HBE Conditions

**Figure 1:**
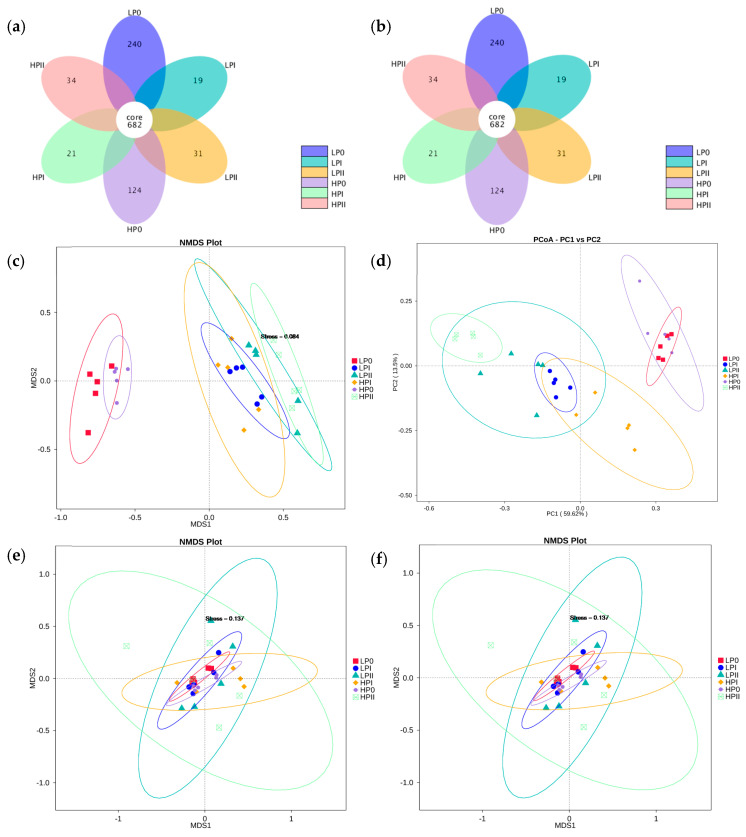
Flower plots of OTUs; (**a**) 16S rRNA and (**b**) 18S rRNA, nonmetric multidimensional scaling analysis for the bacterial communities (**c**) and fungal communities (**e**) based on the Bray–Curtis similarity index, and taxonomic structure of the compost bacterial microbiota (**d**) and fungal microbiota (**f**) at the phylum level.

In order to identify the genetic information of microbial community, a total of 2570 bacterial OTUs and 1730 fungal OTUs were observed in this study. The flower plot displaying bacterial OTUs in the fermentation substrate shows that 682 cores bacterial OTUs (Figure 1a) and 689 core fungal OTUs (Figure 1b) were shared for all composting stages under both the LBE and HBE conditions. Diversity analysis of the LBE and HBE compost samples is shown in Table 1. The observed species in the HBE and LBE conditions were not much different within the same phase of fermentation (*p* > 0.7). The observed species, Chao1 and ACE indices of the microbiota with prolonged composting time in the fermentation phases (PI and PII) were greatly decreased compared to those in P0 (*p* < 0.05). Consistent values of the Shannon and Simpson indices were detected for bacterial diversity among both phases and conditions.

The NMDS plots and PCoA based on weighted UniFrac distances showed that, at the OTU level, the bacterial communities changed more in composition during the fermentation phase than did the fungal communities (Figure 1c–f). The results revealed that the identified taxa of the bacterial communities successfully partitioned the compost samples into two independent groups. The initial phase of composting (P0) under both the LBE and HBE conditions could be distinguished from the fermentation phases (PI and PII). The bacterial communities in these two phases (phases I and II) could not be clearly separated between the LBE and HBE conditions (Figure 1c,d). However, the UPGMA cluster tree based on weighted UniFrac distances showed that the microbiota profiles of PII in HBE were distinct from others (Figure S1a). The results illustrated that altering the fermentation time can influence both the diversity and abundance of bacteria communities. No significant changes in the fungal community composition among the different phases or conditions were detected (Figure 1e,f). The fungal community structures were not distinct among the phases or between the yield conditions. However, the UPGMA cluster tree based on weighted UniFrac distances showed that the fungal profiles of PII in HBE were distinct from others (Figure S1b).

**Figure 2:**
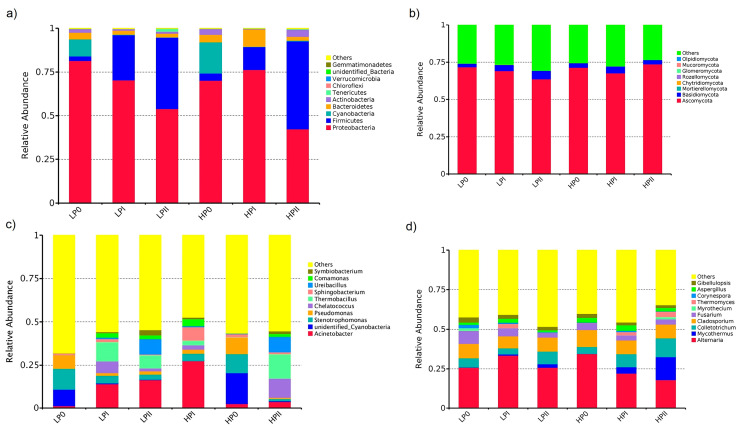
Composition and abundance profiles of the bacterial community (**a**) and fungal community (**b**) at the phylum level and the relative abundance of bacterial communities (**c**) and fungal communities (**d**) at the genus level.

All bacterial sequences were affiliated with the top 10 phyla (Figure 2a). In general, the bacterial communities in the composting process under both conditions were dominated by the following four phyla: *Proteobacteria*, *Firmicutes*, *Cyanobacteria*, and *Bacteroidetes*. *Proteobacteria* was most abundant in P0 but was later replaced by *Firmicutes* as composting progressed under both conditions. The *Proteobacteria* genera *Stenotrophomonas* and *Pseudomonas* were most abundant at the initial phase of composting under both conditions, and their relative abundances decreased over time (Figure 2c). The microbial community present at the end of phase II represents a climax community, in which mushroom mycelia are introduced to the fermented substrate [8]. The abundance of *Firmicutes* (50.3%) under the HBE conditions at PII was greater than that under the LBE conditions (40.8%).

**Figure 3:**
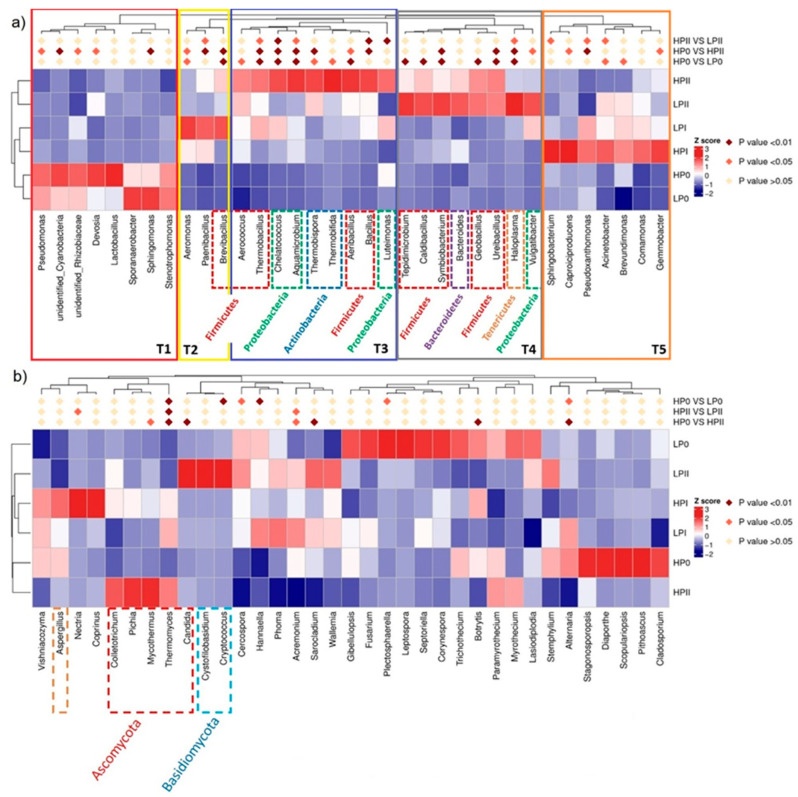
Annotation heatmap of bacterial communities (**a**) and fungal communities (**b**) at the genus level.

Several studies have focused on bacterial communities and their metabolic functions during composting [16,18,20] and revealed that actinomycetes and thermophilic bacteria are dominant and play key roles in compost decomposition. *Firmicutes* have an important effect on cellulose decomposition and substrate utilization, and it was the highest during the thermophilic phase of composting and was universally distributed in the compost [16,38,39]. Some *Firmicutes* can form heat-resistant endospores, and their relative abundance is expected to increase during the thermophilic phase of composting [40]. This study found that the relative abundance of *Firmicutes* in LBE was lower than in HBE. The observed result aligns with the characteristics of carboxymethyl cellulase (CMCase) and xylanase as depicted in Figure S2a,b, where the enzyme activity in HBE was consistently greater than in LBE throughout phase I of composting. The relative abundance of *Proteobacteria* at the end of phase II of LBE composting was higher than that in the HBE compost. Particularly the abundance of the *Vulgatibacter*, a kind of *Proteobacteria* that utilizes cell wall components [41], was considerably higher in LBE compared to HBE (Figure 3a). Guo et al. [16] reported that *Actinobacteria* was the most abundant phylum in the first phase of peach sawdust composting; however, this group of bacteria in the HBE compost was the fourth most abundant phylum in phases I and II of composting in this study. The bacterial community structures in the LBE and HBE composts differed in terms of the relative number but not the group of bacteria. Vieira et al. [18] reported that the abundances of *Proteobacteria*, *Actinobacteria*, and *Bacteroidetes* decreased in composting phase I of sugarcane straw supplemented with wheat bran and then increased gradually in composting phase II. The variations in bacterial community structure profiles may stem from the availability of diverse substrate materials, which have a different microbial community in fermentation systems.

The dynamics of fungal phyla during the composting phase under both productivity yield conditions are shown in Figure 2b. Ascomycota was the most abundant fungal phylum, followed by Basidiomycota. The abundance of Ascomycota increased at the end of composting phase II under the HBE conditions (71.4% to 73.7%) but decreased under the LBE conditions (71.7% to 63.7%). Basidiomycota was more abundant under the LBE condition (5.6%) than under the HBE treatment (2.9%). The fungal community structure differed between the HBE and LBE conditions at the end of fermentation. A greater number of fungal groups was detected in the HBE compost composted substrate than in the LBE compost (Figure 2d). Future work should examine whether basidiomycetes occur at the end of composting phase II as mushroom competitors. Basidiomycetous competitors may suppress mushroom development.

Figure 3a shows a heatmap of the cluster analysis of microbial communities’ phylogenetic structures at the genus level for LP0-II and HP0-II. The genera-clustering tree revealed similarities between samples from the two composts at different stages, dividing the genera into five parts: T1, T2, T3, T4, and T5. The T1 group occupied in HP0 and LP0 and the relative abundance of microorganism decreased as the composting progressed, indicating a similar microbial community structure at the start of the compost. During phase I of composting, the microbial community of LPI was mainly composed of T2 group, followed by the T5 group, while the microbial community of HPI was mainly composed of T5. Both microorganisms in T2 (including *Aeromonas*, *Paenibacillus*, and *Brevibacillus*) and T5 (including *Sphingobacterium*, *Caproiciproducens*, *Pseudoxanthomonas*, *Acinetobacter*, *Brevibacillus*, *Comamonas*, and *Gemmobacter*) can degrade cellulose and lignin, but microorganisms in T2 are better at degrading lignin, while T5 is better at degrading cellulose. Gupta et al. [42] reported that *Aeromonas* was able to remove 78% of lignin. Choudhary [43] revealed that the presence of *Acinetobacter* is notably involved in the degradation of lignin-containing compounds and xenobiotics. In addition, it possesses the ability to scavenge other noxious compounds. However, this study observed low laccase activity during the composting process (Figure S2c), thus other lignin-degrading enzymes, such as lignin peroxidase, may be involved in degradation by *Acinetobacter* during the composting process [44]. At −10 °C, *Sphingomonas* was found to produce CMCase to degrade cellulose [45]. The CMCase content of HPII in Figure S2a is higher than that of LPII, which further proves this point. During phase II of composting, the microbial community of LPII was mainly composed of T4 (such as *Tepidimicrobium*, *Caldibacillus*, *Symbiobacterium*, *Geobacillus*, *Haloplasma*), while the microbial community of HPII was mainly composed of T3 (such as *Thermobacillus*, *Thermobifida*, *Bacillus*), followed by T4. The microorganisms from groups T3 and T4 can degrade cellulose at high temperatures, but HPII has more bacterial species, which is better for substrate maturation. *Bacillus* was found to be associated with metabolism of butanoate and the fermentation of acetyl-CoA into butyrate [46]. The higher relative abundance of Bacillus leads to increased production of butyrate, which in turn lowers the pH of the compost. This observation is consistent with the results shown in Figure S3c. Before composting, substrate raw materials are generally piled up outdoors for several days, where they undergo lignocellulose prefermentation, thereby reducing the duration of subsequent composting [16,47]. The variable types of substrate materials may result in differences in community structures and fermentation process efficiency.

In terms of relative abundance, the significantly dominant fungi in the HBE compost belonged to Ascomycota (*Collectotrichum*, *Pichia*, *Mycothermus*, *Thermomyces*), while the dominant fungi in the LBE compost after phase II of fermentation belonged to Basidiomycota (*Cystofillobasidium*, *Cryptococcus*) (Figure 3b). *Cryptococcus pseudolongus* has been reported to be a pathogen of shiitake mushroom (*Lentinula edodes*). It inhibited the mycelial growth of an *L*. *edodes* strain (cultivar Sanjo 701ho) and caused browning of the mycelia on potato dextrose agar (PDA) [48]. *Mycothermus thermophilus* (Syn. *Scytalidium thermophilum*), a thermophilic fungus, reportedly produces appreciable amounts of cellulases and hemicellulases [21,49]. Cellulase and xylanase, involved in lignocellulosic degradation, were observed throughout the mushroom production cycle in this study, and their activities increased during the fermentation process (Figure S2a,b). Souza et al. [21] reported that *M. thermophilus* and *Thermomyces lanuginosus* were the most abundant species in phase II of composting of sugarcane bagasse and coast-cross hay-based compost for the cultivation of *Agaricus subrufescens*. *Thermomyces lanuginosus* produces various hemicellulases and cellulases during composting [19,50] and has been identified as the dominant species during the thermophilic phase of maize straw composting [19]. Kertesz and Thai [8] reported that *Lewia*, *Rhizomucor*, and *Aspergillus* were the first fungi to grow at the initial phase of composting, followed by overgrowth by *Talaromyces*, *Thermomyces*, and *Thermus*. Noncellulolytic fungi grow by utilizing other carbon sources, not lignocellulose, in compost at the initial phase, and once the alternative carbon sources are consumed, the fungal communities are replaced by cellulose degraders such as *Chaetomium thermophilum* and *Mycothermus thermophilus*.

In this study, *Thermomyces lanubinosus*, *Colletotrichum theobromicola*, *Colletotrichum siamense*, and *Mycothermus thermophilus* were identified as the predominant fungal species at the end of composting phase II under the HBE conditions, while *Colletotrichum truncatum* was identified as the predominant fungal species under the LBE conditions. Salar and Aneja [51] reported the abundances of Hyphomycetes (*Torula thermophila*, *Stibella thermophila*), Ascomycetes (*Chaetomium thermophila*, *Thermoascus aurantiacus*, *Myriococcum albomyces*), and Basidiomycetous species during phase II of composting, and these recovered fungi were almost all cellulolytic fungi, suggesting that they play a role in substrate decomposition. Moreover, they reported that the thermophilic fungi *T*. *thermophila*, *C*. *thermophila*, and *M*. *sulfurea* frequently colonize compost. The thermophilic fungus *T*. *lanuginosus* is known to promote cellulose decomposition when grown in combination with cellulolytic fungi.

### 3.2. Functional Guilds of Microbial Communities in LBE and HBE Conditions

**Figure 4:**
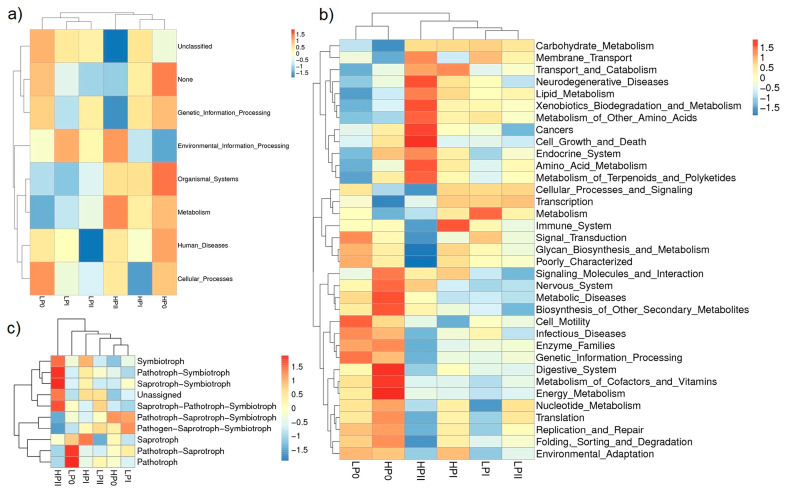
Bacterial functional profile predictions annotated by PICRUSt showing the level 1 KEGG ortholog functional annotation at different phases of composting under the HBE and LBE conditions (**a**). The level 2 KEGG ortholog function prediction annotation of the relative abundance of the 35 most metabolic functions at different composting stages between the two conditions (**b**). Phylogram heatmap illustrating the relative abundances among the treatments across the composting stages; guild annotation using the FUNGuild database (**c**).

In this study, the bacterial functions associated with composting were predicted based on PICRUSt and KEGG pathway analyses. As shown in Figure 4, most of the pathways predicted based on the relatively abundant bacterial sequences with metabolic systems at KEGG level 1 in all the compost samples were related to functions associated with metabolism, environmental information processing, genetic information processing, and cellular processes (Figure 4a). The level 2 KEGG pathway annotations predicted that metabolism under the HBE conditions was stronger than that under the LBE conditions at the initial and final phases of composting (Figure 4b). The functions during in the initial stage of HBE composting mainly included the following metabolic pathways: metabolism of cofactors and vitamins, energy metabolism, metabolic disease, biosynthesis of other secondary metabolites, and cellular processes (digestive system). These pathways had greater relative abundances under the HBE conditions than under the LBE conditions. The relative abundance was obviously different between the two conditions during phase II of fermentation. The functions of the bacterial community during this phase of HBE composting mainly involved the following metabolic pathways: carbohydrate metabolism, lipid metabolism, amino acid metabolism, terpenoid and polyketide metabolism, and xenobiotic biodegradation and metabolism. Furthermore, the fungal co-occurrence network patterns were analyzed (Figure 4c). Fungal network analysis indicated that the abundance of predicted pathotrophs-symbiotrophs and saprotrophs-symbiotrophs gradually increased during HBE composting phase II, while these fungal networks were less abundant in the LBE compost. This finding suggests that enhancing the synergistic interactions among pathotrophs-symbiotrophs and saprotrophs-symbiotrophs may lead to the high yield of this mushroom.

The high relative abundance of amino acid metabolism was consistent with previous results for composting processes based on manure with sugarcane bagasse [22], rice straw [17], and maize straw [20]. The increased abundance of bacteria involved in amino acid metabolism could result in the acceleration of amino acid production [14,17]. The amino acids are required for the metabolic intensity. The abundance of amino acid metabolic functions affects amino acid production and humic substance synthesis [14]. The degradation of hemicellulose and cellulose through carbohydrate metabolism generates various carbohydrate compounds [52]. Therefore, carbohydrate metabolism plays vital roles in lignocellulosic degradation. The growth and proliferation of composting microorganisms in peach sawdust-based composting can be promoted by high total nitrogen levels [16]. The metabolism of terpenoids and polyketides was the most abundant metabolic pathway, followed by carbohydrate metabolism during corncob-based composting [5]. Starch and sucrose metabolism mainly occur in the late stage of corncob composting [5] and swine manure composting [53]. *Bacteroides* played a role in the metabolism of linoleic acid during composting, as evidenced by the increase in lipid biosynthesis proteins. This led to a higher abundance of *Bacteroidetes* during the PI and PII stages of composting [54].

### 3.3. Relationships Between Physiochemical Properties and Microbial Communities

In our previous report [26] describing a comparative study, the physicochemical characteristics, lignocellulose content, and lignocellulosic enzyme activity of the fermented substrate were analyzed during fermentation. The results showed that the average fresh weight and protein content of the fruiting bodies obtained from the LBE conditions was significantly greater than those obtained from the HBE conditions. The ash content of the fruiting body obtained from the LBE conditions was significantly lower than that from the HBE conditions, while the ash content of the compost in LBE was greater than that in HBE as indicated in Figure S3a. Additionally, the electrical conductivity (EC) value of LBE was higher than that of HBE (Figure S2b). These findings corroborated the positive correlation between electrical conductivity and inorganic salt content, which subsequently led to a decrease in the absorption and use of inorganic salt by the mushroom mycelium, resulting in the ash level in the fruiting body of straw mushrooms being lower in LBE compared to HBE. The total sugar content did not differ significantly (*p* < 0.05) (Figure S3c). There was no significant difference in the pH or water content between the two conditions, but the pH continuously decreased during fermentation due to the action of *Bacillus* as described in Section 3.1 (Figure S3d). The ash and nitrogen contents in the substrate gradually increased during the production cycle under both conditions (Figure S3a,e). The C/N ratio during substrate fermentation under the HBE conditions was higher than that under the LBE conditions, and the ratio decreased with fermentation time under both conditions (Figure S3f). Low laccase activity was observed during composting (Figure S2c). The CMCase and xylanase activities in the HBE compost were significantly higher than those in the LBE compost (Figure S2a,b). At the early stage of composting, the presence of more readily utilizable substrate in the compost may cause low levels of lignocellulosic enzyme production, and the degradation increases when the microbial biomass increases and enzyme production begins [55,56]. The lignocellulosic components can be degraded to varying degrees by thermophilic microorganisms during composting [22]. Hemicellulose showed a greater degradation ratio during composting than cellulose and lignin because of its lower molecular weight with short-branched polymers [57,58]. The degradation products of hemicellulose (pentoses and hexoses) are appropriate carbon sources for microbial growth and metabolism during composting [22].

**Figure 5:**
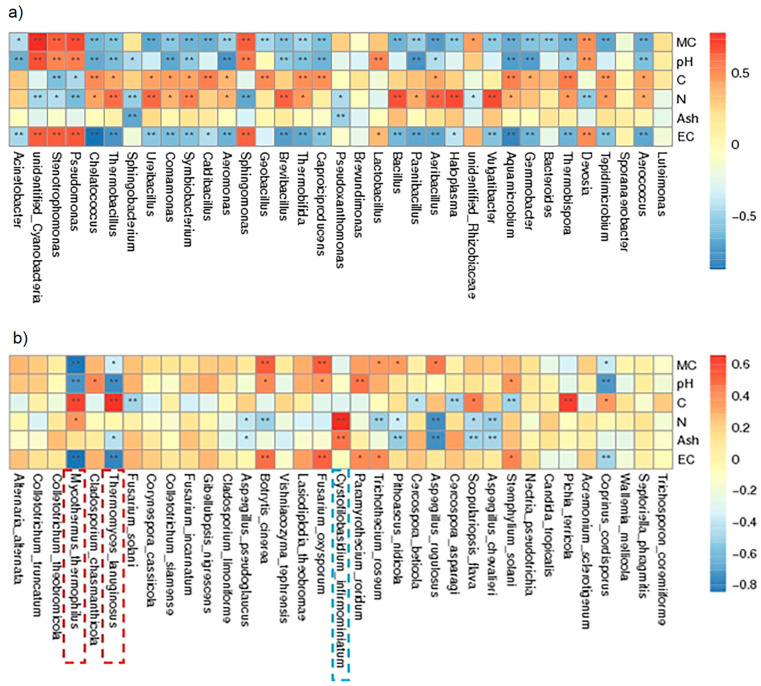
Combined analysis of microbial metabolites and bacterial communities (**a**) and fungal communities (**b**). Red indicates positive correlations between the bacterial community and microbial metabolites, and blue indicates negative correlations between the bacterial community and microbial metabolites. The symbols ** and * indicate that the correlation *p* values were less than 0.01 and 0.05, respectively. MC (moisture content), pH, C (total carbon), N (total nitrogen), Ash, EC (electricity conductivity).

All dominant bacterial genera under both conditions were negatively related to moisture content, electrical conductivity, and pH. At the end of composting phase II, the pH and EC under the LBE conditions were higher than those under the HBE conditions, but the moisture content did not significantly differ between the two conditions. The dominant *Firmicutes* (*Thermobacillus*, *Ureibacillus, Symbiobacterium*, *Brevibacillus*, *Bacillus,* and *Aeribacillus*) were positively related to the nitrogen content. *Proteobacteria* (*Chelatococcus), Firmicutes* (*Caldibacillus*, *Geobacillus),* and *Actinobacteria* (*Thermobifida, Thermobispora*) were positively related to the carbon content. The abundance of *Pseudomonas* was positively related to the EC, and the highest EC value was observed in the initial phase of composting; *Pseudomonas* was one of the dominant genera (Figure 5a). The distributions of Ascomycota, *Thermomyces lanuginosus,* and *Mycothermus thermophilus* were positively related to carbon content and negatively related to pH and EC, while Basidiomycota and *Cystofilobasidium infirmominiatum* were positively related to the nitrogen content. These results corresponded with the carbon and nitrogen contents in composting phase II, in which the carbon content under the HBE conditions was greater than that under the LBE conditions, and vice versa for the nitrogen content (Figure 5b).

## 4. Conclusions

In this study, physiochemical factors (EC, pH, carbon, and nitrogen content) are related to the dominance of microbial communities. Changes in the microbial community and diversity cause different metabolic pathways, which resulted in different biological efficiency of *V*. *volvacea*. The bacteria *Proteobacteria*, with the genus *Chelatococcus*, and *Firmicutes,* with the genus *Thermobacillus*, and the fungi included *Ascomycota* species belonging to the genera *Colletotrichum*, *Pichia*, *Mycothermus*, and *Thermomyces* play a key role in utilization of cellulose and hemicellulose during composting process. In HBE compost, a higher relative metabolic value of bacteria was observed, along with positive correlations between fungal networks and the abundances of both pathogen-symbionts and saprophyte-symbionts. These findings provide key evidence and expand the understanding of microbial communities and their metabolic functions in the conversion of CW for the production of high yields of *V*. *volvacea*.

## Figures and Tables

**Table 1:** Microbial I number and diversity index during composting.

rRNA Gene	Condition	Phase	Observed Species	Chao1	ACE	Shannon	Simson	Good’s Coverage
16S	LBE	P0	1087 ± 124	1239.05 ± 92.63	1276.24 ± 105.01	6.673 ± 0.152	0.964 ± 0.006	0.995 ± 0.000
		PI	814 ± 36	944.44 ± 47.93	949.75 ± 42.92	6.832 ± 0.184	0.971 ± 0.011	0.996 ± 0.000
		PII	803 ± 47	915.66 ± 94.43	934.93 ± 81.19	6.643 ± 0.451	0.966 ± 0.021	0.996 ± 0.001
		P0	960 ± 120	1073.07 ± 116.98	1091.89 ± 126.42	6.951 ± 0.494	0.970 ± 0.022	0.996 ± 0.000
	HBE	PI	831 ± 65	961.12 ± 65.97	976.32 ± 70.13	6.768 ± 0.312	0.972 ± 0.005	0.996 ± 0.001
		PII	813 ± 79	938.89 ± 121.18	939.98 ± 123.18	6.900 ± 0.220	0.976 ± 0.004	0.997 ± 0.001
18S	LBE	P0	713 ± 14	751.80 ± 125.12	758.08 ± 17.20	5.987 ±0. 306	0.921 ± 0.027	0.999 ± 0.000
		PI	677 ± 42	718.87 ± 44.52	714.26 ± 45.63	5.700 ± 0.701	0.876 ± 0.076	0.999 ± 0.000
		PII	572 ± 42	608.98 ± 48.45	612.36 ± 42.74	5.420 ± 0.616	0.886 ± 0.079	0.999 ± 0.000
	HBE	P0	681 ± 49	728.38 ± 40.26	729.48 ± 39.06	5.672 ± 0.520	0.904 ± 0.047	0.999 ± 0.000
		PI	670 ± 117	709.20 ± 113.07	706.34 ± 113.95	6.028 ± 0.239	0.923 ± 0.038	1.000 ± 0.000
		PII	672 ± 144	714.50 ± 146.03	715.50 ± 138.22	5.989 ± 0.458	0.939 ± 0.025	0.999 ± 0.000

## Data Availability

The data presented in this study are available upon the request from the corresponding author, and the sequence data were available on the NCBI, and the access number is PRJNA1213994.

## Supplementary Materials

The following supporting information can be downloaded at: https://www.mdpi.com/article/10.3390/microorganisms13020437/s1, Figure S1: Beta diversity analysis of microbiota structure in compost of HBE and LBE, UPGMA cluster tree based on Weighted UniFrac distance at phylum level (a) bacteria (b) fungi; Figure S2: Production of lignocellulose degrading enzymes during composting and mushroom growing; (a) CMCase, (b) Xylanase and (c) Laccase activity. These data normalized to dry weight biomass, and all data were repeated three times (n = 3), and the results were expressed as mean ± standard deviation (Mean ± SD); Figure S3: Mushroom fruiting body characterizations and physiochemical properties during composting and mushroom growing; (a) ash content, (b) EC value, (c) crude protein and total sugar content, (d) pH, (e) nitrogen content, (f) C/N ratio. These data normalized to dry weight biomass, and all data were repeated three times (n = 3), and the results were expressed as mean ± standard deviation (Mean ± SD).
