# Supplementary material for: Comparison of the Bacterial and Fungal Communities and Metabolic Functions of Cottonseed Hull Waste Compost Associated with High and Low Yields of Straw Mushroom Volvariella volvacea
Source: Microorganisms. 2025 Feb 17;13(2):437. doi: 10.3390/microorganisms13020437 (PMC11858250; doi:10.3390/microorganisms13020437)
Supplement: Supplementary file 1 [file microorganisms-13-00437-s001.zip › microorganisms-3366964-supplementary.pdf]

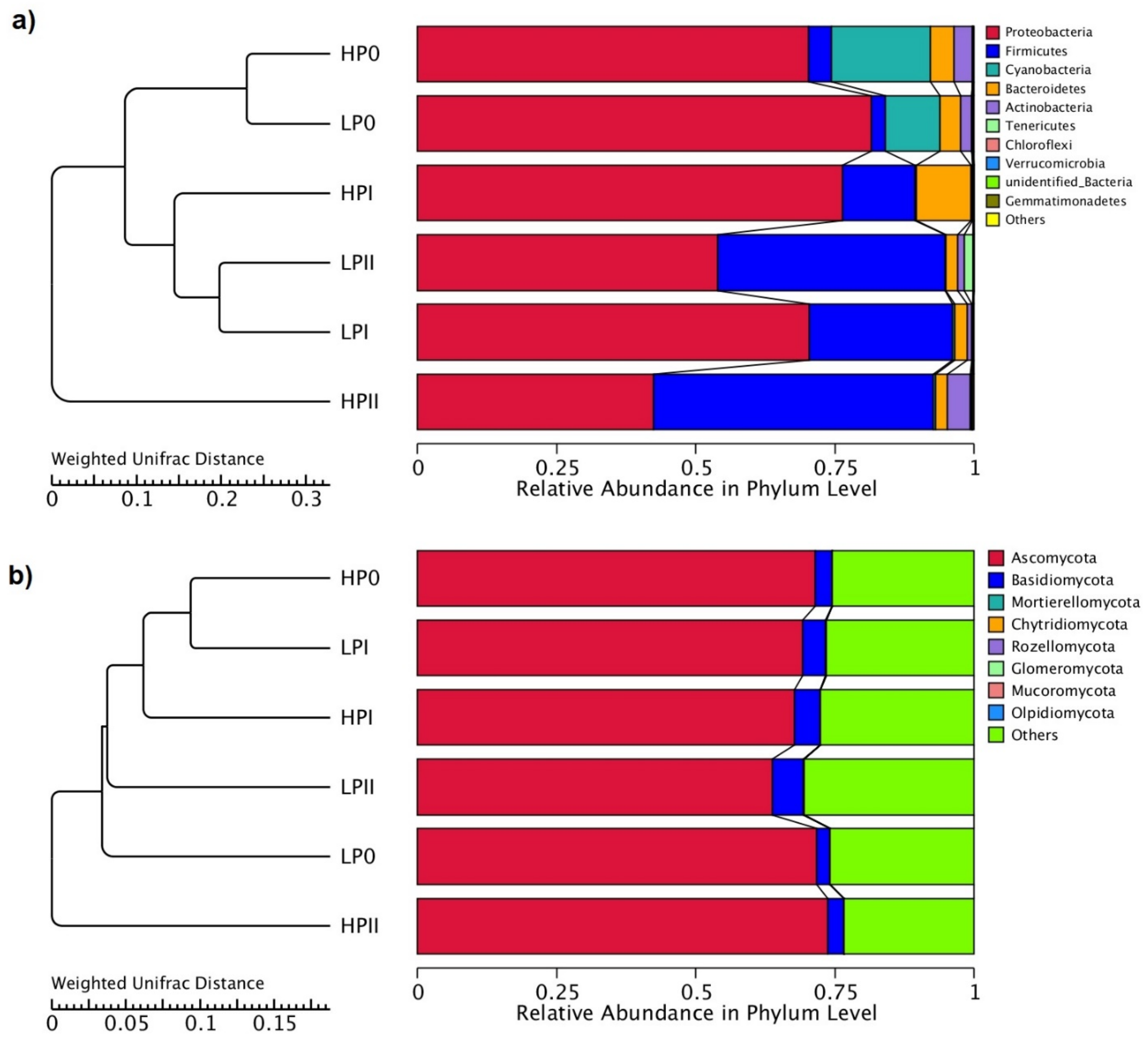

Fig. S1. Beta diversity analysis of microbiota structure in compost of HBE and LBE, UPGMA cluster tree based on Weighted UniFrac distance at phylum level (a) bacteria (b) fungi.

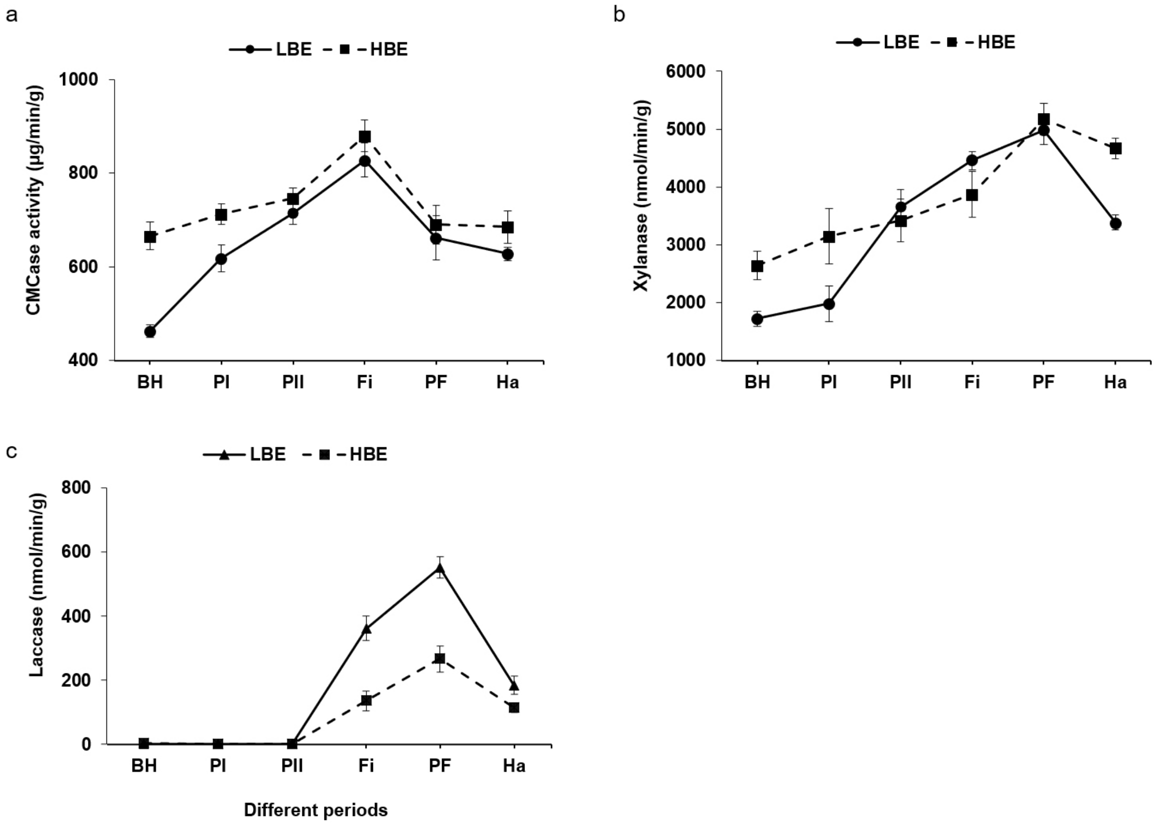

Fig. S2. Production of lignocellulose degrading enzymes during composting and mushroom growing; a) CMCase, b) Xylanase and c) Laccase activity. These data normalized to dry weight biomass, and all data were repeated three times (n=3), and the results were expressed as mean ± standard deviation (Mean ± SD).

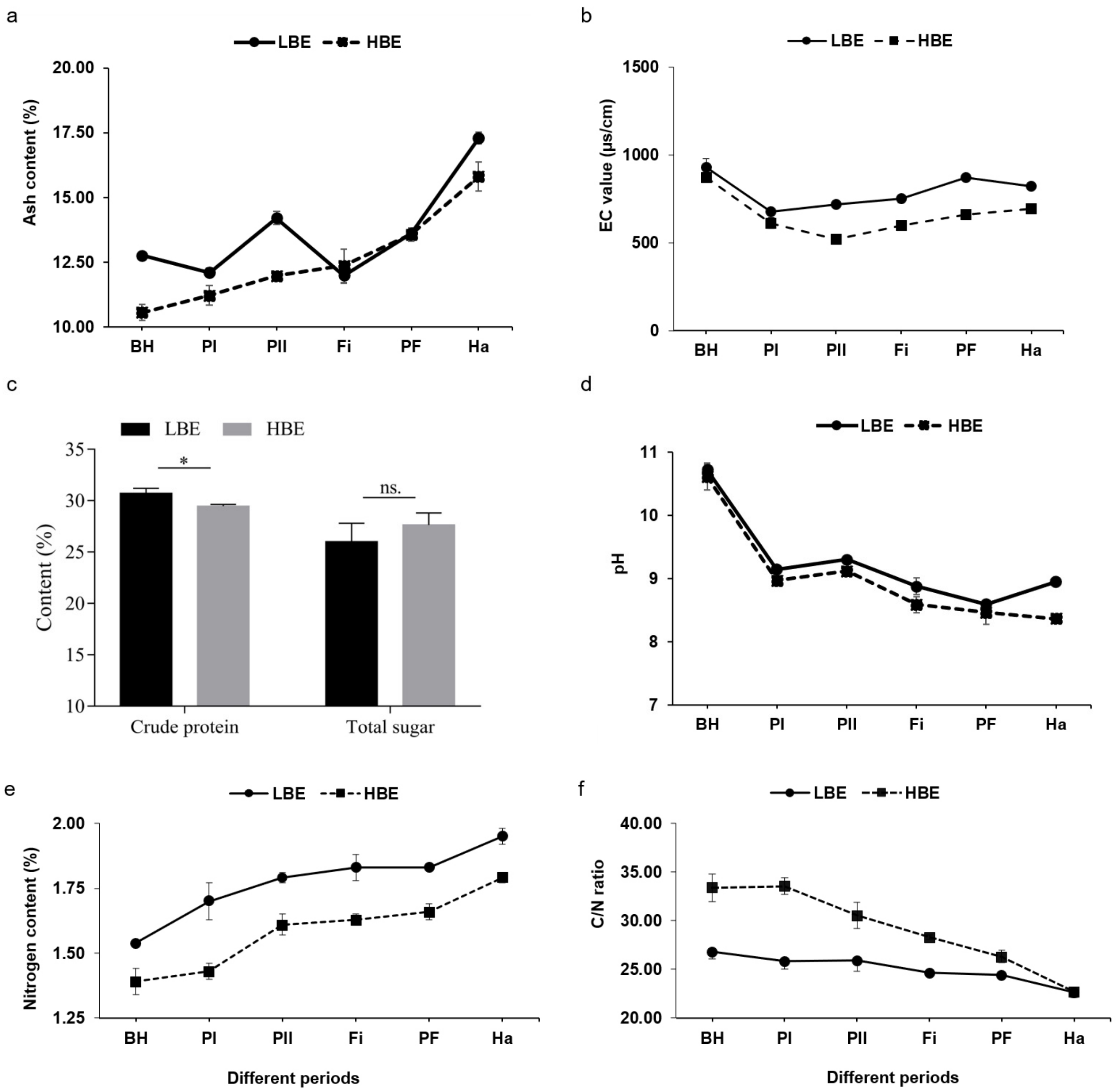

Fig. S3. Mushroom fruiting body characterizations and physiochemical properties during composting and mushroom growing; a) ash content, b) EC value, c) crude protein and total sugar content, d) pH, e) nitrogen content, f) C/N ratio. These data normalized to dry weight biomass, and all data were repeated three times (n=3), and the results were expressed as mean ± standard deviation (Mean ± SD).
